# Mortality Following *Clostridioides difficile* Infection in Europe: A Retrospective Multicenter Case-Control Study

**DOI:** 10.3390/antibiotics10030299

**Published:** 2021-03-13

**Authors:** Jacek Czepiel, Marcela Krutova, Assaf Mizrahi, Nagham Khanafer, David A. Enoch, Márta Patyi, Aleksander Deptuła, Antonella Agodi, Xavier Nuvials, Hanna Pituch, Małgorzata Wójcik-Bugajska, Iwona Filipczak-Bryniarska, Bartosz Brzozowski, Marcin Krzanowski, Katarzyna Konturek, Marcin Fedewicz, Mateusz Michalak, Lorra Monpierre, Philippe Vanhems, Theodore Gouliouris, Artur Jurczyszyn, Sarah Goldman-Mazur, Dorota Wultańska, Ed J. Kuijper, Jan Skupień, Grażyna Biesiada, Aleksander Garlicki

**Affiliations:** 1 Department of Infectious and Tropical Diseases, Jagiellonian University Medical College, 30-688 Krakow, Poland; gbiesiada@op.pl (G.B.); agarlicki@gmail.com (A.G.); 2 Department of Medical Microbiology, Charles University, 2nd Faculty of Medicine and Motol University Hospital, 15006 Prague, Czech Republic; marcela.krutova@lfmotol.cuni.cz or; 3 ESCMID Study Group for Clostridioides Difficile (ESGCD), 4001 Basel, Switzerland; amizrahi@hpsj.fr (A.M.); nagham.khanafer@chu-lyon.fr (N.K.); hanna.pituch@wum.edu.pl (H.P.); e.j.kuijper@lumc.nl (E.J.K.); 4 Service de Microbiologie Clinique, Groupe Hospitalier Paris Saint-Joseph, 75014 Paris, France; lorra.monpierre@hotmail.fr; 5 Institut Micalis UMR 1319, Université Paris-Saclay, INRAe, AgroParisTech, 92290 Châtenay Malabry, France; 6 Unité d’Hygiène, Epidémiologie et Prévention, Groupement Hospitalier Centre, Hospices Civils de Lyon, 69002 Lyon, France; philippe.vanhems@chu-lyon.fr; 7 Centre International de Recherche en Infectiologie (CIRI), Public Health, Epidemiology and Evolutionary Ecology of Infectious Diseases (PHE3ID), Université de Lyon, 69372 Lyon, France; 8 Clinical Microbiology & Public Health Laboratory, Public Health England, Addenbrooke’s Hospital, Hills Road, Cambridge CB2 0QQ, UK; david.enoch@addenbrookes.nhs.uk (D.A.E.); theodore.gouliouris@addenbrookes.nhs.uk (T.G.); 9 Hygienic Department, Bács-Kiskun County Teaching Hospital, 6000 Bács-Kiskun, Hungary; patyim@kmk.hu; 10 Department of Propaedeutics of Medicine and Infection Prevention, Ludwik Rydygier Collegium Medicum in Bydgoszcz, Nicolaus Copernicus University, 85-094 Bydgoszcz, Poland; deptula.aleksander@gmail.com; 11 Department of Medical and Surgical Sciences and Advanced Technologies “GF Ingrassia”, University of Catania, 95123 Catania, Italy; agodia@unict.it; 12 Critical Care Department, Vall d’Hebron Hospital, Vall d’Hebron Institut de Recerca (VHIR), SODIR Group, Universitat Autònoma de Barcelona, 08035 Barcelona, Spain; fxnuvials@vhebron.net; 13 Department of Medical Microbiology, Medical University of Warsaw, 02-004 Warsaw, Poland; dorota.wultanska@wum.edu.pl; 14 Department of Internal and Geriatric Diseases, Jagiellonian University Medical College, 30-688 Krakow, Poland; mwojcik@su.krakow.pl; 15 Department of Pain Treatment and Palliative Care, Jagiellonian University Medical College, 30-688 Krakow, Poland; inusia_bryniarska@yahoo.pl; 16 Department of Gastroenterology and Hepatology, Jagiellonian University Medical College, 30-688 Krakow, Poland; bartek.brzozowski@op.pl; 17 Department of Nephrology and Dialysis Unit, Jagiellonian University Medical College, 30-688 Krakow, Poland; mkrzanowski@op.pl; 18 Intensive Care Unit, University Hospital, 30-688 Krakow, Poland; kliber@su.krakow.pl; 19 Józef Babiński Hospital, 30-393 Krakow, Poland; marcin.fedewicz@gmail.com; 20 Ludwik Rydygier Hospital, 31-826 Krakow, Poland; michalakcontact@gmail.com; 21 Department of Hematology, Jagiellonian University Medical College, 30-688 Krakow, Poland; mmjurczy@cyf-kr.edu.pl (A.J.); senoritagoldman@gmail.com (S.G.M.); 22 Department of Medical Microbiology, Centre for Infectious Diseases, Leiden University Medical Center, 2333 Leiden, The Netherlands; 23 Centre for Infectious Diseases Research, Diagnostics and Laboratory, Surveillance, Rijksinstituut voor Volksgezondheid en Milieu, 2333 Bilthoven, The Netherlands; 24 Department of Metabolic Diseases, Jagiellonian University Medical College, 30-688 Krakow, Poland; jan.skupien@gmail.com

**Keywords:** *Clostridioides difficile* infection, co–morbidities, mortality, malignancy, outcome, risk factors

## Abstract

We aimed to describe the clinical presentation, treatment, outcome and report on factors associated with mortality over a 90-day period in *Clostridioides difficile* infection (CDI). Descriptive, univariate, and multivariate regression analyses were performed on data collected in a retrospective case-control study conducted in nine hospitals from seven European countries. A total of 624 patients were included, of which 415 were deceased (cases) and 209 were still alive 90 days after a CDI diagnosis (controls). The most common antibiotics used previously in both groups were β-lactams; previous exposure to fluoroquinolones was significantly (*p* = 0.0004) greater in deceased patients. Multivariate logistic regression showed that the factors independently related with death during CDI were older age, inadequate CDI therapy, cachexia, malignancy, Charlson Index, long-term care, elevated white blood cell count (WBC), C-reactive protein (CRP), bacteraemia, complications, and cognitive impairment. In addition, older age, higher levels of WBC, neutrophil, CRP or creatinine, the presence of malignancy, cognitive impairment, and complications were strongly correlated with shortening the time from CDI diagnosis to death. CDI prevention should be primarily focused on hospitalised elderly people receiving antibiotics. WBC, neutrophil count, CRP, creatinine, albumin and lactate levels should be tested in every hospitalised patient treated for CDI to assess the risk of a fatal outcome.

## 1. Introduction

*Clostridioides difficile* (*C. difficile*), formerly known as *Clostridium difficile*, is the most common cause of healthcare-associated infectious diarrhoea in the developed world. The incidence and severity of *C. difficile* infections (CDI) have risen in recent years with a considerable impact in terms of morbidity, mortality, and financial cost [1]. The burden of healthcare-associated CDIs in acute care hospitals in the European Economic Area was estimated at 123,997 cases annually [2]. In the United States, *C. difficile* is the most common cause of healthcare–associated infections, accounting for approximately 15% of them [3]. According to data from 2012, *C. difficile* caused approximately half a million infections and 29,000 deaths in the US [4]. The pooled incidence rate of CDI in Asia was calculated by meta-analysis at 5.3/10,000 patient days (95% CI 4.0–6.7) [5].

Increasing antibiotic use, improved life expectancy, increasing numbers of at-risk patients and the emergence of hypervirulent epidemic strains (e.g., ribotype PCR 027) may explain the increased incidence of CDI and these factors, in addition to hospitalisation, are key factors in the development of CDI [6,7]. Inflammatory bowel disease, gastrointestinal surgery, and conditions impairing the immune system (e.g., malignant neoplasms, transplantation, chronic kidney disease, and immunosuppressant use) also predispose towards CDI [8,9]. The clinical spectrum of CDI varies in severity from asymptomatic carriage and self-limited, mild diarrhoea to severe colitis, intestinal perforation, toxic megacolon, and death [7,10]. Mortality rates in CDI vary widely between studies. Before 2000, mortality associated with CDI was <2%, whereas mortality in studies since 2000 averaged 5% in endemic case and 7–17% in epidemic cases [11,12,13,14,15]. The mortality has been twice as high in Intensive Care Unit (ICU) patients with CDI compared to ICU patients without CDI [16,17].

We aimed to describe the risk factors, clinical presentation, and management of patients with CDI as well as reported factors associated with mortality in the 90-day period after diagnosis.

## 2. Materials and Methods

### 2.1. Study Population

Using the European Society of Clinical Microbiology and Infectious Diseases (ESCMID) Study Group for C. difficile members, 17 hospitals were selected for this study. Nine hospitals from seven European countries (Czech Republic, France, Hungary, Italy, Poland, Spain, and United Kingdom) participated in this retrospective case-control study.

Patients hospitalised between January 2011 and December 2019 with a diagnosis of CDI who died within 90 days following a CDI diagnosis formed the case group that was compared in a 2:1 ratio to a group of control patients with a CDI diagnosis hospitalised in the same wards over the same time period who survived.

### 2.2. Data Collection

Electronic hospital databases were used to collect patient data on: sex, age, body mass index (BMI), prior hospitalisations, dwelling in a long-term care (LTC) facility, recent surgery, parenteral nutrition, previous use (in last 3 months) of antibiotics, probiotics, proton pump inhibitors (PPIs), H2 blockers (H2b), immunosuppressants (defined as agents that can suppress or prevent the immune response), information on comorbidities needed to calculate the Charlson Index and dates of admission, CDI diagnosis, and deaths. The following data on the CDI episode were gathered: episode number, blood parameters at the time of diagnosis [white blood cell count (WBC), neutrophil count, C-reactive protein (CRP), creatinine, albumin, and lactate levels], associated bacteraemia, imaging procedures performed (abdominal ultrasound, computed tomography), colonoscopy and CDI therapy, as well as outcomes and complications (i.e., failure of any organ, infection, ileus, colon perforation, toxic megacolon, and bleeding from the digestive tract). 

### 2.3. Definitions

A CDI case was defined as a patient with the symptoms of CDI and positive laboratory test(s) according to ESCMID guidelines [18]. Healthcare–associated CDI (HA–CDI) was defined as a patient who developed the symptoms of CDI in a healthcare facility on day three or later, following admission to a healthcare facility on day one, or who had onset in the community within four weeks after being discharged from a healthcare facility. Community-associated CDI (CA–CDI) was defined as a patient who had the onset of symptoms either outside of the healthcare facilities, or whose symptoms appeared in a healthcare facility within 48 h after admission but who had not been discharged from a healthcare facility within a 12 week period prior to the onset of symptoms [19]. Cases that did not fit any of these criteria were classified as unknown.

### 2.4. Statistical Analysis

For descriptive purposes continuous variables are presented as medians, lower (1 st) and upper (3 rd) quartiles. Categorical variables are presented as counts and percentages. Summary statistics were computed for the group of deceased patients and the control group of patients, who recovered from CDI. The frequencies of categorical variables were compared with the χ^2^ test or Fisher’s exact test, where applicable. Spearman correlation coefficients with appropriate asymptotic tests were calculated for select continuous variables and time to death. For categorical variables (including binary and ordinal ones), we used Kendall’s correlation. To identify a set of statistically independent predictors of CDI mortality, we used logistic regression models. Variable selection was performed using the LASSO (least absolute shrinkage and selection operator) method with 10-fold cross-validation. A one standard deviation rule was used to select a parsimonious set of candidate variables. In the final multivariate logistic model, we retained only statistically significant predictors, and their joint predictive performance was evaluated with C-statistic. *p*-values <0.05 were considered to be significant. Data processing and statistical calculations were performed with R 3.6.3 (The R Foundation for Statistical Computing, Vienna, Austria).

## 3. Results

### 3.1. Descriptive Analysis of Included Patients

Data were collected from 624 hospitalised patients with CDI; 415 patients died (cases) and 209 patients were still alive 90 days after a CDI diagnosis (controls). The gender distribution was similar in both groups but slightly skewed toward women, (210; 51% in the deceased group), versus 118 (57%) in the controls (*p* = 0.17). The median age was 80 years in the deceased group and 72 years in the control group (*p* < 0.001). People ≥ 65 years-old constituted 86% (n = 357) of the deceased group and 67% (n = 140) in the control group (*p* < 0.001). People ≥80 years-old constituted 50% (n = 208) of the deceased group and 28% (n = 59) in the control group (*p* < 0.001). The patients’ median age in the deceased group was, on average, 8 years higher and their Charlson Index was twice as high as in the control group (*p* < 0.001). The comparison of data on previous hospitalisations, surgeries, LTC stays, parenteral nutrition, use of probiotics, PPI, and H2b between the two groups of patients are shown in Table 1.

The number of patients who had HA-CDI was 280 (68%) among the deceased patients and 131 (63%) in the control group with CA-CDI. Fourteen (3%) in the deceased group and 20 (10%) in the control group (*p* = 0.006) were classed as CA–CDI. The origin of the CDI was unknown in 121 patients (29%) from the deceased group and in 58 patients (28%) from the control group.

Table 2 lists the most commonly used antibiotics (or antibiotic class) in the preceding 3 months. The use of β-lactams was most prevalent in both groups. The administration of fluoroquinolones was more frequent in the deceased group than in the control group. 

The incidence of co-morbidities as possible risk factors for CDI mortality is shown in Table 3. At least one co-morbidity was recorded in 76% of patients in the deceased group and 53% in the control group (*p* < 0.001). In the deceased group, a malignancy was the most prevalent comorbidity. Diabetes, chronic kidney disease, cachexia, and liver cirrhosis were also more common in deceased patients compared to controls.

### 3.2. The Clinical Course of CDI

Data pertaining to the course of CDI are shown in Table 4. An increased WBC, neutrophils, CRP, creatinine, and lactate, and a lower serum albumin concentration were found to be significantly more frequent in patients with a fatal outcome of CDI compared to the control group. Complications during the course of CDI and cognitive impairment were more common in the deceased group compared to controls. The time between admission of patients and a CDI diagnosis was longer in the deceased group of patients compared to the controls.

Abdominal ultrasound was performed in 36 cases in the deceased group (9%) and 37 cases (18%) in the control group (*p* < 0.001). Findings of importance in CDI (ascites, thickening of the intestinal wall of the colon, and/or increase in the lumen diameter of the colon) were discovered in 29 cases (81%) of the deceased group, compared to 14 (38%) in tests performed on the control group (*p* < 0.001). CT imaging was performed in 54 cases in the deceased group (13%) and in 15 cases in the control group (7%), *p* = 0.03. Important findings on CT (same as in ultrasonography) were discovered in 76% of procedures performed on the deceased group, compared to 67% in the control group (*p* = 0.51). Colonoscopy was performed on 7 patients in the deceased group (1.7%) and on 5 patients in the control group (2.4%), *p* = 0.55.

Fourteen different CDI treatment regimens were employed in the deceased group and eight in the control group. The chosen therapy was changed due to side effects in 3 cases (1%) in the deceased group, and in 3 cases (1%) in controls. Oral metronidazole was the most common treatment in both groups, n = 179, at 43% in the deceased group, and n = 100, as well as 48% in the control group. This treatment was unchanged in 131 and 86 patients from the respective groups. Thirteen people (3.1%) in the deceased group and 5 patients (2.4%) in the control group did not receive any antibiotic treatment for CDI.

The most common complications in the deceased group were failure of at least one vital organ (n = 36; 9%), pneumonia (n = 32; 8%), and sepsis (n = 14; 3%). Blood cultures were positive in five patients in the deceased group; the confirmed pathogens were: *Acinetobacter baumannii*, *Citrobacter koseri*, *Enterobacter cloacae*, *Candida albicans* and a mixture from one patient (*Staphylococcus epidermidis*, *Staphylococcus haemolyticus,* and *Enterococcus faecium*). Complications involving the gastrointestinal tract included ileus (n = 7; 2%), toxic megacolon (n = 3; 1%), bleeding (n = 3; 1%), intestinal ischaemia (n = 2; 0.5%), and gastrointestinal perforation (n = 1; 0.2%). The most commonly occurring complications in the control group were secondary infections: pneumonia (n = 10; 5%), urinary tract infections (n = 6; 3%), and sepsis (n = 4; 2%).

### 3.3. Predictors of Death in CDI

Multivariate logistic regression identified 11 factors that together discriminated CDI deaths from controls. The most important were advanced age, the presence of malignancy, a higher Charlson Index, WBC (1000/μL increase), CRP (100 mg/L increase), the presence of complications, and the presence of cognitive impairment (Table 5). The discriminative accuracy of this model was considerably high, as the C–statistic was 0.864.

Correlation was assessed between selected parameters and the time from CDI diagnosis to death. Advanced age, higher levels of WBC, neutrophil, CRP or creatinine, the presence of malignancy, cognitive impairment, and complications were strongly correlated with hastening death (Table 6).

Research data are available as Supplementary Materials.

## 4. Discussion

To the best of our knowledge, this study is the largest analysis of patients with a fatal outcome of CDI in multiple sites across Europe. 

We confirm that increasing age is an important risk factor for a fatal outcome of CDI, as described previously [20,21]. A systematic review which included 30 studies showed that increasing age is among the most reported risk factor for mortality in patients with CDI [22]. This is most likely due to the weaker immune response to the *C. difficile* toxin. Moreover, the elderly are characterized by a greater number of chronic diseases, including those that contribute to a worse course of CDI, such as chronic kidney disease, diabetes, or malignancy [23]. Gender is not an important factor since the risk was similar for both males and females, as described in other studies [21,24,25].

The presence of any comorbidity (*p* < 0.0001) and increasing Charlson Index (*p* = <0.0001) were also associated with increased mortality. This has been described elsewhere [20,21,26], but some studies did not confirm such observations [27]. This may be the result of the different characteristics of the population or study design. Oncology patients face a number of risk factors that are predisposed to CDI acquisition, including frequent and prolonged hospitalisations, increased antibiotic use (both prophylactic and therapeutic), and chemotherapy [28]. We found malignancy to be an independent factor of mortality risk. The pathogenesis of CDI, during and after chemotherapy, is not yet fully understood but suggestions include a negative impact on the gastrointestinal microbiota, direct damage to intestinal mucosa, and immunological mechanisms in the neoplastic process predisposing to CDI [29,30,31]. In a large analysis of outcomes of 30,000 patients with cancer, those with CDI had a significantly higher mortality rate (9.4% vs. 7.5%, *p* < 0.0001) [32]. Among other comorbidities, liver cirrhosis and cachexia were more prevalent in the deceased group but only cachexia was independent death risk predictor. Patients with liver cirrhosis and cachexia are typically characterised by low levels of albumin, a recognised risk factor for severe CDI [7], and in our study, low albumin levels were strongly correlated with the risk of death. Surprisingly IBD was not related with 90-days mortality; however, the total number of patients with IBD in both groups was low. IBD is a known risk factor both for development of CDI and mortality [24,33]. We also noted an association with chronic kidney disease and increased mortality which has also been reported previously, particularly in patients with end-stage renal disease and patients on dialysis, compared to the general population [34,35]. Diabetes is also a predisposing factor for CDI development and recurrence [36,37], but it was not shown to be a mortality-related factor [20,25,27]. In our study diabetes was more prevalent in the deceased group, but it was not independent death predictor, when assessed in the multivariate logistic regression model.

CDI is a recognised problem in LTC facilities; residents are often elderly with multiple co-morbidities. LTC admission 90 days before CDI was related with 30-day all-cause mortality in one study [27]. We also found that previous LTC residency was more prevalent in the deceased group and was independently associated with a fatal outcome.

It is suggested that a patients’ weight has an impact on outcome from CDI. One study suggested that being underweight (BMI < 19) or morbidly obese (BMI > 40) was associated with an increased in-hospital mortality in patients with CDI [38], while another reported that underweight patients with CDI are at higher risk of poor outcome than normal, overweight, and obese patients [39]. Therefore, one of our aims was to establish if there was any association between a patient’s BMI and its impact on the course of CDI. Calculating BMI sometimes poses a challenge, since the most severely ill, often bedridden patients cannot be weighed properly. BMI data were only available in 55% of our cases; however, considering the large population of patients in our study, their number was sufficient to conclude that BMI did not increase mortality in CDI.

Previous hospitalisation was more prevalent in the deceased group but was not independent risk factor of death, which confirmed the findings of Morrison et al. study [24].

In our study, the previous use of PPIs and/or H2b before a CDI episode was not associated with a fatal outcome; these findings are contrary to a study by Morrison et al. [24]. In another report, the use of PPIs, but not H2b, was a predictor of mortality within 30 days after the end of treatment for a CDI recurrence; however, there are some differences in the methodology compared to our study [27].

Antibiotic use alters gut microbiota that physiologically protects the gastrointestinal tract from colonisation by pathogens, including *C. difficile*. In our study, we demonstrated that antibiotic treatment was the most common risk factor for CDI mortality (92% in the deceased group and 88% in the control group). The most frequently used antimicrobials were β-lactams and fluoroquinolones, two of the “4C” antibiotics in which stewardship intervention can lead to a decline in prevalence of epidemic *C. difficile* ribotypes [40].

In our population of 624 study participants, every patient had at least one risk factor for the development of CDI. It is also notable that only 7 patients (1.1%) did not have any of the 3 main CDI risk factors (age > 65; previous hospitalisation or antibiotic use). This is of importance, since it suggests that patients with no risk factors are less likely to develop CDI.

We analysed blood parameters which are known risk markers for poor outcomes in CDI [41,42,43]. These parameters are very useful in clinical practice, as they can be assessed cheaply, objectively, and early in the course of CDI. The differences in their values between both groups are pronounced. It is worth noting, however, that although the WBC count was almost always tested, this was not always the case for other parameters and the percentage of tests performed (creatinine, CRP, neutrophil count, albumin and lactate) was low. We found that WBC and neutrophil counts, CRP, and creatinine were strongly correlated with shortening the time from CDI diagnosis to death. Moreover, WBC and CRP were independent predictors of death.

In the deceased group, 88% of patients died during the first episode which is consistent with the notion that the highest risk of death is associated with the first episode of CDI [44]. Colonoscopies were rarely performed in our study. Endoscopic evaluation can be useful; however, it is indicated only if diagnostic problems occur, e.g., when an alternative diagnosis is suspected and direct visualisation and/or biopsy of the bowel mucosa is needed [7]. Computed tomography and ultrasounds are useful among patients with severe CDI helping to evaluate for presence of complications like toxic megacolon or bowel perforation [45]. The number of these examinations in our study was relatively small, and it is especially surprising that ultrasound examinations were performed much less frequently in the deceased group.

Oral metronidazole was the most frequently used drug in CDI treatment in our study. This is despite recent guidance suggesting vancomycin and/or fidaxomicin be used as first line in CDI [10]; however, the majority of patients in our study were hospitalised before this guideline could be implemented. Nevertheless, most patients did not receive the correct treatment choice according to the guidelines [10,18]. This indicates the need for hospitals’ infection prevention and control teams to organise dedicated seminars on CDI for medical personnel. The knowledge about correct antibiotic prescriptions and antimicrobial resistance is one of the main important threats identified by the World Health Organisation [46]. One study involving 1179 junior doctors found that questions on antimicrobial use were poorly answered, whilst 81% of participants stated that teaching about appropriate antimicrobial use was inadequate during their medical training and 71% disagreed that they received the right examples from their tutors [47].

Our study shows that CDI therapy was well tolerated, since the percentage of patients whose therapy was altered due to side effects was very small. As it can be seen in Supplementary Materials, almost all patients were treated with the use of well-known conventional drugs. Only one patient was a participant of a cadazolid trial. Cadazolid is a novel quinoxolidinone antibiotic developed for treating CDI, which was safe and well tolerated but did not achieve its primary endpoint of non-inferiority to vancomycin for clinical cure in one of two phase 3 CDI trials [48]. Three patients were treated with the use of intravenous immunoglobulin (IVIG) which sometimes is used in CDI treatment, but reports as to its effectiveness are ambiguous [49,50]. None were treated with Bezlotoxumab, a monoclonal antibody that binds to *C. difficile* toxin B, which was approved by the FDA in 2016 for prevention of recurrent CDI in patients at high risk of CDI recurrence [51].

Our study has several limitations. It was a retrospective study and some data were unavailable. In addition, there are substantial differences in the numbers of patients included by each centre. However, we did not want to refuse any centre that wished to participate. We were unable to distinguish between cases when CDI was the primary cause of death and when it was not, and there was no ribotyping / sequencing data.

## 5. Conclusions

In our multicentre study, the independent risk factors for mortality at day 90 were older age, inadequate CDI therapy, cachexia, malignancy, Charlson Index, LTC facility care, elevated WBC, elevated CRP, bacteraemia, complications, and cognitive impairment. CDI prevention should be primarily focused on hospitalised elderly people receiving antibiotics, especially fluoroquinolones or β-lactam/β-lactamase inhibitors. For this group, we suggest using available preventive measures all the time, instead of, as is presently often done, after CDI diagnosis.

## Figures and Tables

**Table 1:** Comparison of demographic and clinical data in the study groups.

Characteristic	CDI–Deceased Group (N = 415)	CDI–Control Group (N = 209)	*p* Value
	N (%) or Median (1st, 3rd Quartile)	N (%) or Median (1st, 3rd Quartile)	
Age (years)	80 (70, 86)	72 (59, 82)	<0.0001
Sex (male)	205 (49.4%)	91 (43.5%)	0.17
BMI (kg/m^2^) *	24.2 (21.1, 27.7)	25.0 (22.1, 27.8)	0.39
Charlson Index	4 (3, 6)	2 (1, 4)	<0.0001
Previous hospitalisations	313 (75.4%)	132 (63.2%)	0.001
Previous parenteral nutrition	33 (8.0%)	17 (8.1%)	0.94
Previous surgery	77 (18.6%)	47 (22.5%)	0.24
Previous LTC facility	56 (13.5%)	10 (4.8%)	0.0008
Previous probiotics use	61 (14.7%)	21 (10.0%)	0.11
Previous PPI use *	219 (56.4%)	104 (50.2%)	0.15
Previous H2b use *	22 (5.3%)	9 (4.3%)	0.59

BMI, body mass index; CDI, *Clostridioides difficile* infection; H2b, H2 blockers; LTC, long term care; PPI proton pomp inhibitors.* Missing data: BMI: 186 cases in the deceased group and 49 cases in the control group; PPI use: 27 cases in the deceased group and 2 cases in the control group; H2b use: 2 cases in the deceased group and 1 case in the control group.

**Table 2:** Antibiotics used in the 3 months prior to the episode of CDI.

Antibiotic	CDI–Deceased Group N (%)	CDI–Control Group N (%)	*p* Value
No antibiotic	35 (8.4%)	25 (12.0%)	0.16
Fluoroquinolones	160 (38.6%)	51 (24.4%)	0.0004
BLBLI	152 (36.6%)	60 (28.7%)	0.049
Third generation cephalosporins	147 (35.4%)	60 (28.7%)	0.09
Carbapenems	68 (16.4%)	31 (14.8%)	0.62
Aminoglycosides	45 (10.8%)	14 (6.7%)	0.09
Metronidazole	27 (6.5%)	27 (12.9%)	0.007

BLBLI, β-lactam/β-lactamase inhibitor; CDI, *Clostridioides difficile* infection; Some patients used more than one antibiotic, therefore the percentage sum does not equal 100%.

**Table 3:** The frequency of co-morbidities regarded as possible risk factors for CDI mortality.

Comorbidity	CDI–Deceased Group N (%)	CDI–Control Group N (%)	*p* Value
At least one comorbidity	316 (76.1%)	111 (53.1%)	<0.0001
Malignancy	155 (37.3%)	40 (19.1%)	<0.0001
Diabetes mellitus	120 (28.9%)	38 (18.2%)	0.004
Chronic kidney disease	112 (27.0%)	32 (15.3%)	0.001
Immunosuppressive therapy	73 (17.6%)	39 (18.7%)	0.74
Cachexia	47(11.3%)	5 (2.4%)	0.0001
Immunosuppressive disease	17 (4.1%)	6 (2.9%)	0.44
Liver cirrhosis	14 (3.4%)	0 (0%)	0.004
IBD	5 (1.2%)	4 (1.9%)	0.49

CDI, *Clostridioides difficile* infection; IBD, inflammatory bowel disease. Some patients had more than one factor, therefore the percentage sum does not always equal 100%.

**Table 4:** Data pertaining to the clinical course of CDI.

Characteristic	CDI–Deceased Group		CDI–Control Group		*p* Value
	N (%) or Median (1st, 3rd Quartile)	N Missing	N (%) or Median (1st, 3rd quartile)	N Missing	
Body temperature (°C)	36.6 (36.5, 37.1)	127	36.8 (36.6, 37.6)	74	0.005
WBC count (×1000/µL)	13.7 (8.8, 22.3)	14	9.6 (7.2, 14.0)	1	<0.0001
Neutrophil count (×1000/µL)	10.4 (6.1, 18.0)	186	7.2 (4.7, 10.9)	51	<0.0001
CRP (mg/L)	116 (70, 198)	67	65 (23, 120)	17	<0.0001
Serum creatinine (µmol/L)	107 (67, 192)	34	75 (60, 113)	5	<0.0001
Serum albumin (g/L)	24 (20, 28)	173	28 (23, 32)	99	<0.0001
Serum lactate (mmol/L)	1,8 (1.4, 3.3)	356	1.0 (0.8, 1.7)	187	0.0002
Bacteremia	45 (10.8%)	0	6 (2.9%)	0	0.0006
Episode number 1 2 3 4 5	365 (88.0%) 41 (9.9%) 6 (1.4%) 2 (0.5%) 1 (0.2%)	0	177 (84.7%) 22 (10.5%) 5 (2.4%) 5 (2.4%) 0 (0%)	0	0.18
Complications (without deaths)	101 (24.3%)	0	21 (10.0%)	0	<0.0001
Cognitive impairment	92 (22.2%)	0	6 (2.9%)	0	<0.0001
Surgery after CDI diagnosis	10 (2.4%)	0	4 (1.9%)	0	0.78
Days from admission to diagnosis	7 (1, 18)	1	5 (1, 13)	0	0.01
Days from diagnosis to death	12 (4, 25)	0	NA	NA	NA

CDI, *Clostridioides difficile* infection; CRP, C-reactive protein; NA, not applicable; WBC, white blood cells.

**Table 5:** List of independent death predictors in a multivariate logistic regression model.

Covariate	Odds Ratio (95% Confidence Interval)	*p* Value
Age (10–year increase)	1.57 (1.31, 1.89)	<0.001
inadequate antibiotics *	3.70 (1.08, 12.69)	0.04
Cachexia	5.00 (1.34, 18.57)	0.02
Malignancy	2.62 (1.43, 4.81)	0.002
Charlson Index (1 unit increase)	1.24 (1.11, 1.39)	0.0001
long term care	2.42 (1.05, 5.58)	0.04
WBC (1000/μL increase)	1.03 (1.01, 1.06)	0.005
CRP (100 mg/l increase)	1.80 (1.34, 2.43)	0.0001
Bacteremia	3.35 (1.06, 9.93)	0.04
Complications (without deaths)	3.95 (2.08, 7.50)	<0.001
Cognitive impairment	7.50 (2.73, 20.66)	<0.001

Model C-statistic = 0.864; CRP C-reactive protein; WBC white blood cell; *- use of ineffective treatment such as intravenous metronidazole or vancomycin in monotherapy, or ineffective antibiotics, such as tigecycline.

**Table 6:** Spearman or Kendall (for categorical, binary or ordinal variables) correlations between select characteristics and days from CDI diagnosis to death.

Variable	ρ/τ	*p* Value
Age	−0.15	0.0024
Malignancy	0.08	0.038
WBC count	−0.17	0.0005
Neutrophil count	−0.30	<0.0001
CRP	−0.27	<0.0001
Serum creatinine	−0.18	0.0005
Complications (without deaths)	0.11	0.0093
Cognitive impairment	−0.14	0.0008

CRP C–reactive protein; WBC white blood cell, included only variables with *p* < 0.05.

## Data Availability

Research data are available as Supplementary Materials.

## Supplementary Materials

The following are available online at https://www.mdpi.com/2079-6382/10/3/299/s1 Research data.
